# Impurity-Governed Modification of Optical and Structural Properties of ZrO_2_-Based Composites Doped with Cu and Y

**DOI:** 10.1186/s11671-017-1920-4

**Published:** 2017-02-27

**Authors:** N. Korsunska, M. Baran, I. Vorona, V. Nosenko, S. Lavoryk, X. Portier, L. Khomenkova

**Affiliations:** 1 V. Lashkaryov Institute of Semiconductor Physics of National Academy of Sciences of Ukraine, 45 Pr. Nauky, Kyiv, 03028 Ukraine; 2 NanoMedTech LLC, 68 Antonovycha Str, Kyiv, 03680 Ukraine; 3 CIMAP Normandie Univ, ENSICAEN, UNICAEN, CEA, CNRS, 6 Boulevard Marechal Juin, Caen, 14050 France

**Keywords:** Cu-doped Y-stabilized ZrO_2_, Electron paramagnetic resonance, Attenuated total reflection, Diffuse reflectance, Transmission electron microscopy

## Abstract

The influence of calcination temperature on copper spatial localization in Y-stabilized ZrO_2_ powders was studied by attenuated total reflection, diffuse reflectance, electron paramagnetic resonance, transmission electron microscopy, electron energy loss, and energy-dispersive X-ray spectroscopies. It was found that calcination temperature rise in the range of 500–700 °C caused the increase of copper concentration in the volume of ZrO_2_ nanocrystals. This increase was due to Cu in-diffusion from surface complexes that contained copper ions linked with either water molecules or OH groups. This copper in-diffusion led also to an enhancement of absorption band peaked at ~270 nm that was ascribed to the formation of additional oxygen vacancies in nanocrystal volume. Further increasing of calcination temperature from 800 up to 1000 °C resulted in outward Cu diffusion accompanied by a decrease of the intensity of the 270-nm absorption band (i.e., oxygen vacancies’ number), the transformation of ZrO_2_ tetragonal (cubic) phase to monoclinic one as well as the enhancement of absorption band of dispersed and crystalline CuO in the 600–900 nm range.

## Background

Zirconia nanopowders have attracted considerable attention due to their mechanical, electric, thermal, and optical properties offering diverse applications such as catalysts [1], high temperature and corrosion resistant coatings [2], chemical sensors [3–5], radiation detectors [6], biological labeling [7], switchable mirrors or filters [8], etc. Pure and/or Y-stabilized ZrO_2_ (YSZ) demonstrate different emission bands in visible spectral range that make such materials suitable for white light-emitting devices [9–11]. The YSZ ceramics exhibit specific characteristics such as superplasticity at low temperatures. Besides, the YSZ shows high ionic conductivity at elevated temperatures [12, 13], high chemical inertness, thermal stability, and hardness [14] that allowed these materials to be used as electrolytes in solid oxide fuel cells [13, 15] and thermal barriers [16].

In recent years, ZrO_2_ nanocomposites containing other components, in particular CuO, are intensively developed. The properties of such composites depend on Cu spatial localization [17–26]. Copper can be present inside the YSZ grains and/or on their surface (in the form of aggregates of Cu atoms [17], CuO molecules, or crystalline CuO [19, 23]). Copper on the grains’ surface or in its near-surface region was accepted to be responsible for catalytic activity of the composite, its fungicidal properties [17–21], and tribological behavior [16]. Besides, surface Cu location shows also ability to compact the ceramics [24, 25].

Copper, located inside the grains, affects structural characteristics of the composites. Specifically, depending on the calcination temperature and Cu concentration, the monoclinic ZrO_2_ structure can be transformed into tetragonal or cubic [27]. In addition, Cu-doping results in appearance of specific photoluminescence (PL) band in the green spectral range [22] that can be interesting for applications in light-emitting devices.

It should be noted that the influence of copper impurity on the structural characteristics of the composites, as well as their catalytic properties, was extensively studied for monoclinic ZrO_2_ powders (see for example [18, 19, 28]). At the same time, among different ZrO_2_ phases, tetragonal phase is known to exhibit better catalytic properties [29, 30].

To form tetragonal ZrO_2_ phase, the doping with yttrium is often used. However, in comparison with Cu-doped ZrO_2_, the structural properties of (Cu,Y)-codoped ZrO_2_ and the spatial distribution of the impurities in it can differ. For example, Cu addition to Y-doped ZrO_2_ lowers the temperature of tetragonal to monoclinic phase transformation [25]. Besides, it was proposed that in such composite, the formation of Y cuprate on the crystallite surface occurred at elevated temperatures [25].

It should be noted also that most studies were performed on Cu–ZrO_2_ powders doped by impregnation technique that led to predominant localization of copper on the surface of nanocrystals or in their near-surface region. At the same time, coprecipitation technique can allow the Cu presence both in the crystallite volume and at their surface simultaneously. Therefore, the investigation of the effect of technological conditions on Cu spatial localization in ZrO_2_-based composites fabricated by coprecipitation technique keeps interest in terms of the development of the materials with required properties.

In the present paper, (Cu,Y) codoped ZrO_2_ nanopowders prepared by coprecipitation method and calcinated at different temperatures were studied by infrared attenuated total reflection, diffuse reflectance, electron paramagnetic resonance, transmission electron microscopy, electron energy loss, and energy-dispersive X-ray spectroscopy (imaging modes) techniques to obtain information about the dependence of copper spatial localization on the calcination temperature.

## Methods

### Sample Preparation

The Y-stabilized ZrO_2_ nanopowders codoped with Cu were synthesized by a coprecipitation technique using Zr, Y, and Cu nitrates in molar ratio ZrO(NO_3_)_2_:Y(NO_3_)_3_:Cu(NO_3_)_2_ = 96:3:1 and 89:3:8 in distilled water. With such a composition, the CuO concentration of 1 mol% (Cu-1 samples) or 8 mol% (Cu-8 samples) was obtained, whereas the Y_2_O_3_ content was about 3 mol% for both sets.

The 6 wt% ammonia solution was used for a chemical precipitation. The latter was carried out at pH = 10–11 followed by precipitate washing to pH = 7. After washing with distilled water and filtering, the microwave irradiation (700W, 2.45GHz) was used to dry the precipitates. It is worth to note that this method allowed obtaining the powders with uniform distributions of dopants.

Xerogel nanopowders were calcinated at *T*_c_ = 500–1000 ^o^C for 2 h and slowly cooled with the furnace to room temperature.

### Material Characterization

Infrared attenuated total reflection (ATR) spectra were acquired at room temperature using IRAffinity-1 Fourier transform spectrometer equipped with a DTGS (deuterated triglycine sulfate) detector. The Golden Gate single reflection diamond ATR top-plate MKII accessory equipped with ZnSe lenses was used for ATR measurements. The spectra were collected with a mirror speed value of 2.8 mm/s by averaging 32 scans at a resolution of 2 cm^−1^ over the 550–4000 cm^−1^ range. In our experiment, the small amount of powder was pressed on diamond surface by a sapphire anvil. These effects were corrected by using advanced ATR correction.

Diffuse reflectance spectra were recorded with respect to the BaSO_4_ standard at room temperature by means of double-beam spectrophotometer UV-3600 UV-VIS NIR (Shimadzu Company) equipped with an integrated sphere ISR-3100. Obtained spectra were transformed in absorption ones using standard program based on the Kubelka-Munk ratio $f\left({r}_{\infty}\right)=\frac{{\left(1-{r}_{\infty}\right)}^2}{2{r}_{\infty }}=\frac{K}{S}$, where *f*(*r*_∞_) is the Kubelka-Munk function, ${r}_{\infty }={R}_{\mathrm{sample}}/{R}_{{\mathrm{BaSO}}_4}$ is the relative diffuse reflection from the sample, *K* and *S* are absorption and scattering coefficients of the sample, respectively.

Electron paramagnetic resonance (EPR) measurements were carried out using X-band EPR spectrometer Varian E12 (~9.5 GHz) with a sensitivity limit of about 10^12^ EPR centers. A 100-kHz modulation of the magnetic field with peak-to-peak amplitude modulation of 0.1 mT and microwave power of about 2 mW, being much less than the saturation power, were used. The signal of a MgO:Mn sample containing 3 × 10^15^ spins was used as a reference. EPR spectra were normalized with respect to the intensity of the signal of MgO:Mn reference as well as on the mass of each powder studied. All measurements were carried out at room temperature.

For transmission electron microscopy (TEM) observations, powders were mixed to butanol before being spread on a holey carbon copper grid. Conventional TEM observations were performed using a 2010 FEG JEOL microscope operated at 200 kV. High-resolution TEM, STEM High Angle Annular Dark Field (HAADF), energy-filtered TEM images as well as EDX chemical maps were recorded with a double-corrected cold FEG ARM200 JEOL microscope operated at 200 kV and equipped with a Centurio EDX JEOL setup. This latter microscope is also equipped with a Gatan Imaging Filter (QUANTUM 965 ER) from which energy-filtered (EFTEM) images (chemical maps) were produced.

## Results and Discussion

### ATR Spectra

**Fig. 1:**
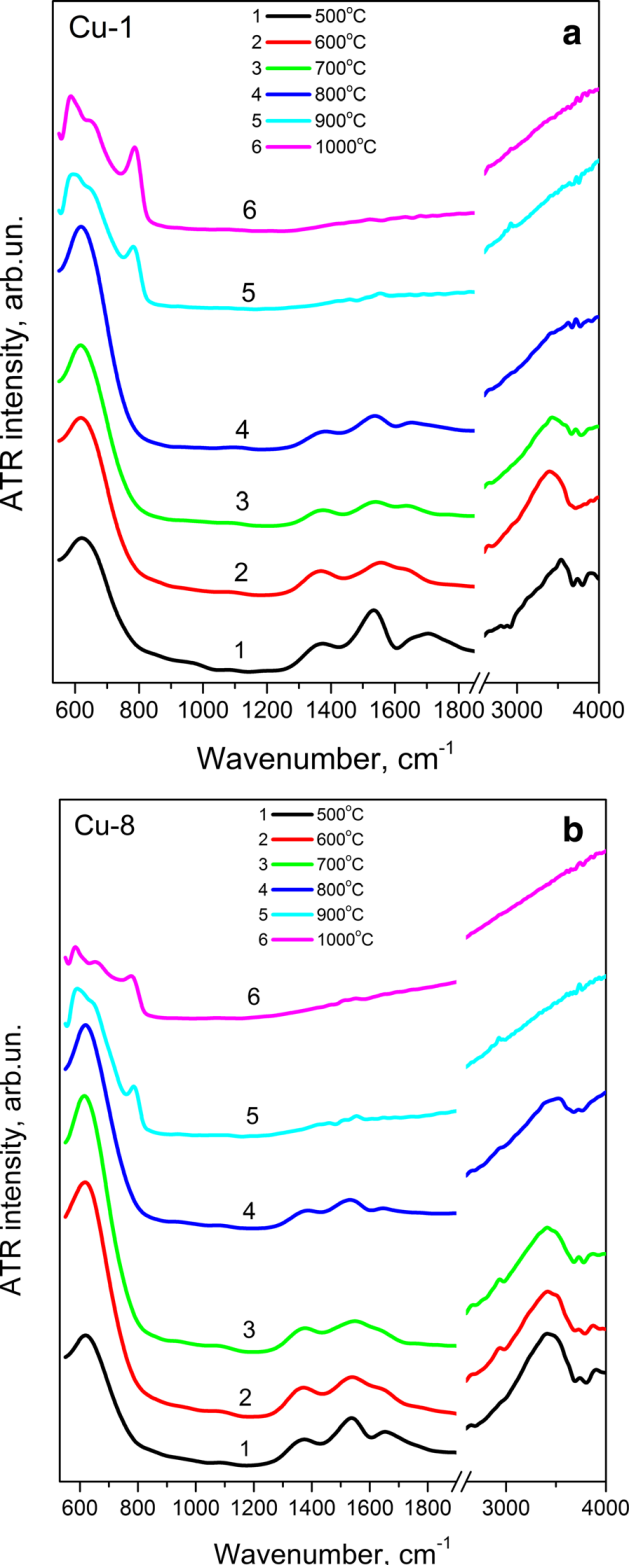
ATR spectra of Cu-1 (**a**) and Cu-8 (**b**) samples calcinated at 500–1000 °C

ATR spectra of Cu-1 and Cu-8 samples calcinated at 500–1000 °C are shown in Fig. 1. These spectra demonstrate Zr–O vibrations in the range of 500–850 cm^−1^. The broad band at ~615–625 cm^−1^ is the major feature in the spectra of the samples for both groups annealed at *T*_c_ ≤ 800 °C (Fig. 1a, b). This band can be attributed to the absorption band of ZrO_2_ lattice with tetragonal and/or cubic phases [31]. The *T*_c_ increase up to 900 °C leads to the appearance of other bands centered at ~575 and ~770 cm^−1^ that are the features of ZrO_2_ monoclinic phase [32]. Its contribution increases with *T*_c_ rise up to 1000 °C. This finding agrees with XRD results [22, 31]. However, XRD patterns show appearance of monoclinic phase with a contribution being approximately 3% already at *T*_c_ = 800 °C [22]. Obviously, such amount of monoclinic phase is insufficient for its detection by ATR technique.

Besides, ATR spectra show also the bands in the 1300–1400 cm^−1^, 1500–1580 cm^−1^, 1580–1680 cm^−1^, and 3000–3700 cm^−1^ spectral regions. The band at 3000–3700 cm^−1^ can be caused by vibrations of OH groups or water molecules [33]. Absorption at 1580–1680 cm^−1^ is attributed to deformation vibrations of adsorbed water. The band at 1500–1580 cm^−1^ is assigned to the deformation vibrations of OH groups bonded with the metal (δ(MOH)), and the band at 1300–1400 cm^−1^ is determined by hydroxyl groups strongly bonded between themselves by hydrogen bonds and structured by hydroxyls of water (γ(OH)) [34].

The intensity of all these bands decreases noticeably with *T*_c_ increase and at *T*_c_ ≥ 900 °C the bands at 3000–3700 cm^−1^ and 1580–1680 cm^−1^ disappear. Since the intensities of these two absorption bands decrease with *T*_c_ synchronously and somewhat faster than the intensities of other two bands (at 1500–1580 cm^−1^ and 1300–1400 cm^−1^), one can assume that not only the band at 1580–1680 cm^−1^, but also the band at 3000–3700 cm^−1^ can be attributed to the vibrations of adsorbed water. Note that according to Ref.[35], water contained in the xerogel evaporates at ~200 °C (during drying), while the main amount of OH groups is lost at *T*_c_ = 400–500 °C. However, Fig. 1 shows the presence of OH groups in our samples calcinated at higher temperatures. This fact can be explained by water adsorption on the nanocrystal surface upon calcination process.

### Diffuse Reflectance Spectra

**Fig. 2:**
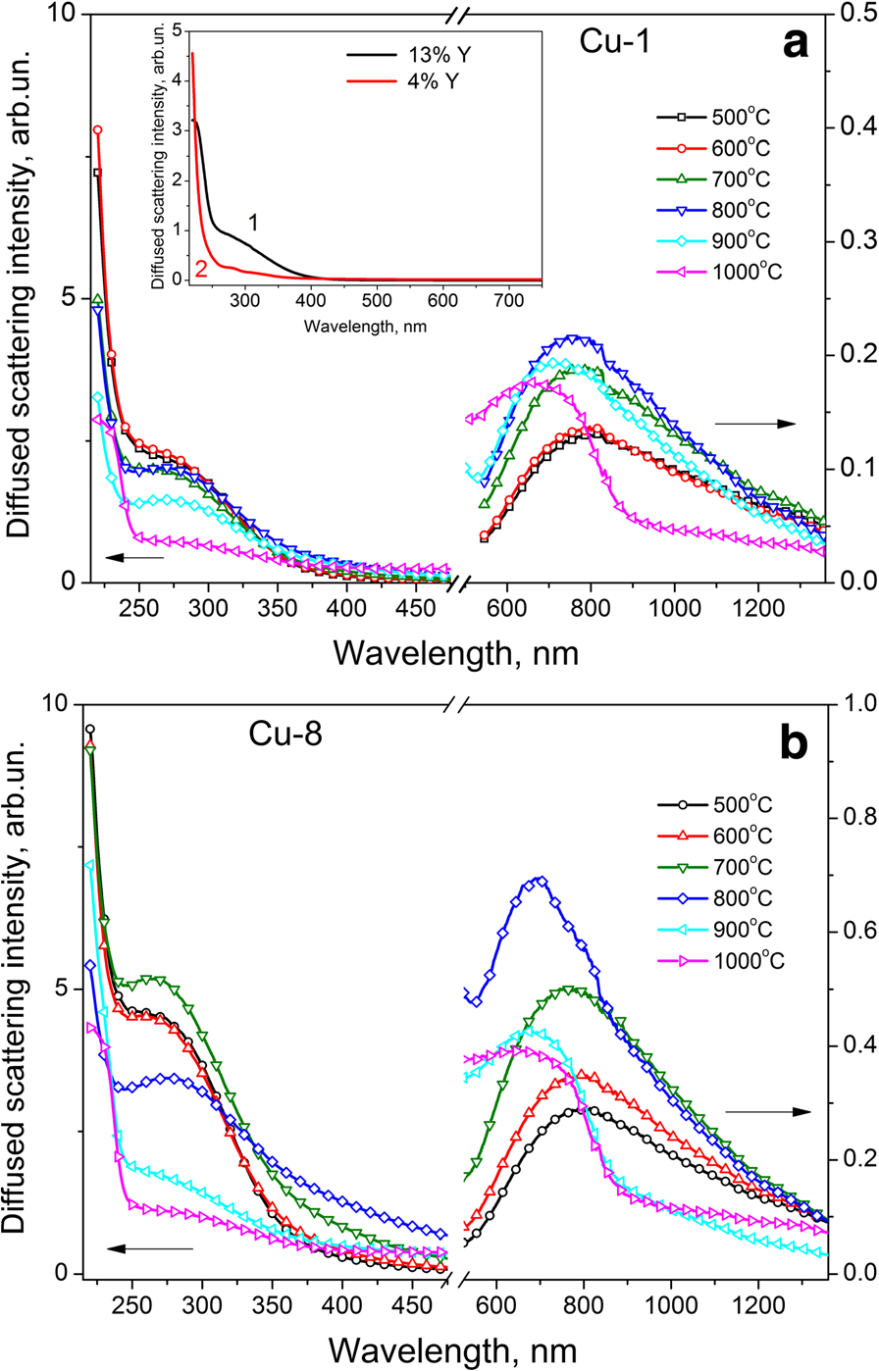
Diffuse reflectance spectra of Cu-1 (**a**) and Cu-8 samples (**b**), calcinated at *T*_c_ = 500–1000 °C, as well as the spectra of Cu-free ZrO_2_ samples doped with 13% of yttrium (*curve 1*) and 4% of yttrium (*curve 2*), *T*_c_ = 700 °C (*inset* in Fig. 2a)

Figure 2 shows diffuse reflectance (DR) spectra of Cu-1 (Fig. 2a) and Cu-8 (Fig. 2b) samples calcinated at different temperatures. These DR spectra contain the absorption band (peaked at ~270 nm) near the band edge of ZrO_2_. Increase of *T*_c_ in the 500–700 °C range leads to a slight increase of its intensity in Cu-8 samples, while it is almost unchanged for Cu-1 samples. For *T*_c_ = 800–1000 °C, the intensity of this band decreases essentially in both groups of samples. Note, that the band at ~270 nm is more intense for Cu-8 samples.

In addition to absorption near ZrO_2_ band edge, the band in the range of 600–900 nm is observed in DR spectra. Its intensity increases with *T*_c_ rise up to 800 °C, being accompanied by the shift of its peak position to shorter wavelengths. For higher calcination temperatures, the edge of the fundamental absorption of crystalline CuO appears for Cu-8 samples (Fig. 2b).

The band in the 600–900 nm range is usually attributed to *d-d* transitions of the Cu^2+^ ions in an octahedral or tetragonal distorted octahedral surrounding [36, 37] and associated with dispersed CuO on the surface of the nanocrystals [36] or with Cu_Zr_ substitutional atoms located in the near-surface region [19, 35]. The increase of Cu loading caused the increase of intensity and the short-wavelength shift of this Cu-related band. The latter was attributed to an increase of octahedral distortion [35, 36].

Based on the obtained experimental data, we can consider the nature of absorption band at ~270 nm. Similar band was observed in the works devoted to study of monoclinic and tetragonal ZrO_2_ doped with Cu by impregnation. The intensity of this band increased with Cu loading, and it was ascribed to electron transitions from copper to oxygen [23, 35].

However, as can be seen from Fig. 2a (inset), the same absorption band is present in the ZrO_2_ samples doped with yttrium only, and its intensity increases with Y concentration. The common feature of both Y- and Cu-doped ZrO_2_ is the formation of oxygen vacancies [36], which are required for charge compensation due to difference in the valences of these impurities and Zr. It allows assigning the band at ~270 nm observed in our samples to oxygen vacancies. Indeed, the intensity of this band decreases at *T*_c_ ≥ 800 °C (Fig. 2) that correlates with the appearance and increase of the contribution of monoclinic phase (Fig. 1) containing less oxygen vacancies than Y- or Cu-stabilized tetragonal phase [36]. It should be noted that in Cu–ZrO_2_ samples doped by impregnation, the Cu atoms can penetrate in the near-surface region of ZrO_2_ nanocrystals [23] and even form solid solution [19] that also should create oxygen vacancies [35]. Therefore, in that case, the oxygen vacancies can also contribute to absorption band at ~270 nm.

The contribution of Cu-surface substances is more pronounced for the samples calcinated at higher *T*_c_. One of the possible candidates can be CuO, whose formation was revealed by diffuse reflectance spectra and detected by XRD method for the similar powders [32].

### TEM Observations

**Fig. 3:**
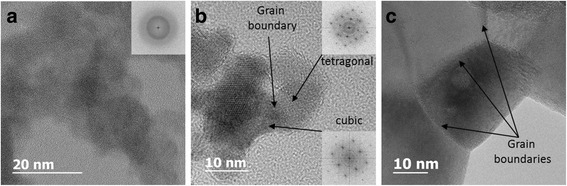
High-resolution TEM images for Cu-8 samples. **a** Xerogel with amorphous grains. **b** *T*_C_ = 600 °C with nanoscale grains. **c** *T*_C_ = 900°C with larger grains

TEM study was performed for both types of samples. However hereafter, only the results for Cu-8 samples are presented because they demonstrate more pronounced variations of their optical properties with *T*_C_. Figure 3 shows the evolution of the structure of the Cu-8 samples with *T*_C_. It is seen that xerogel sample has an amorphous structure. The *T*_C_ increase results in the formation of nanocrystals, whose mean size increases from ~14 nm (*T*_C_ = 600 °C) to ~46 nm (*T*_C_ = 900 °C). For *T*_C_ = 600 °C, the nanocrystals were found to be with tetragonal and cubic structures (Fig. 3b), while for *T*_C_ = 900 °C, the main part of nanocrystals was of monoclinic phase (Fig. 3c). Besides, large particles consisting of smaller nanocrystals with grain boundaries were found. The presence of tetragonal and cubic grains in Cu-8 samples calcinated at 900 °C were also detected, but their amount was much lower.

**Fig. 4:**
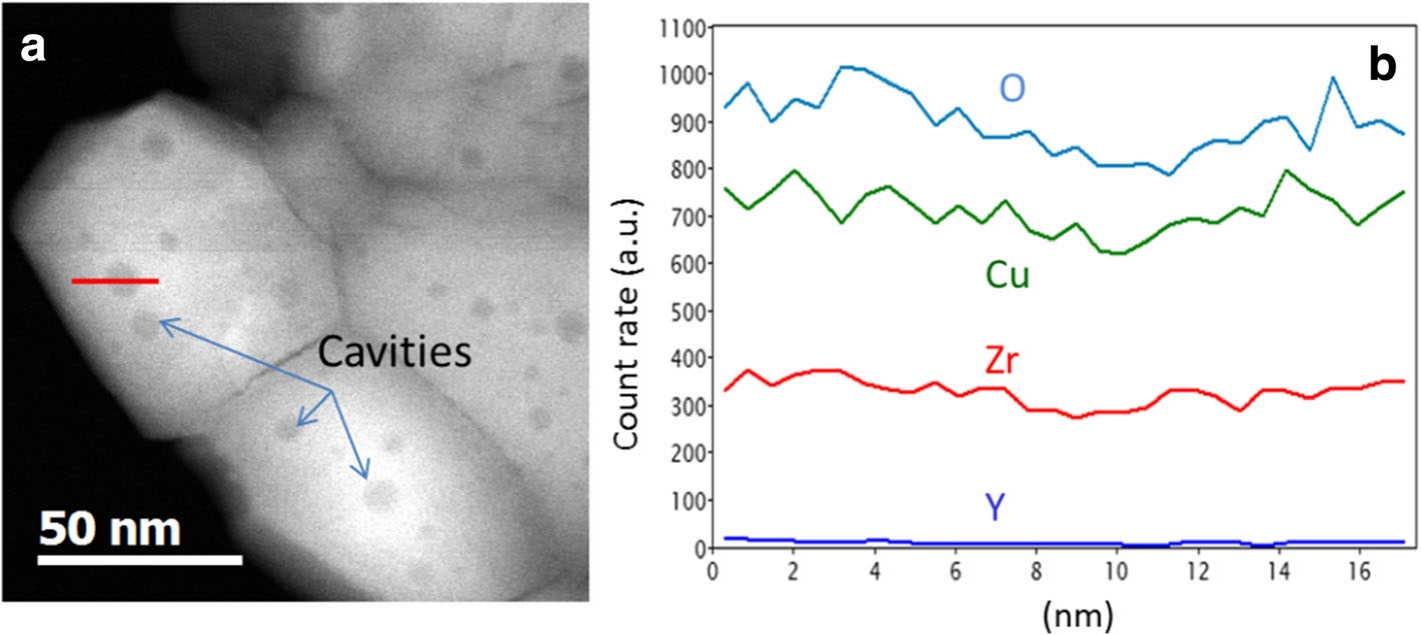
**a** STEM HAADF image of Cu-8 powder calcinated at 900 °C. The darker regions with circular shapes in the grains correspond to cavities. **b** EDX profiles across one of these cavities (*red line in a*) for O, Cu, Y, and Zr elements. A depletion of the number of counts is observed for all the elements

Figure 3c demonstrates the appearance of some circle-like regions within many grains. Chemical analyses have been carried out on these regions. STEM HAADF image, presented in Fig. 4a, shows clearly these darker circular regions in the ZrO_2_ grains. The darker contrast in this observation mode corresponds to a lower mean *Z* value.

The EDX profile across one of these regions (Fig. 4b) indicates a depletion of the count rates for all elements. It is worth to note that the Cu signal is higher than that of Zr despite the low content of Cu compared to that of Zr. This is due to the use of a holey carbon copper grid for powder observation which enhances artificially the copper content in the copper quantification. Anyway, these results are in agreement with the presence of empty cavities. One of the reasons of their formation can be the segregation of vacancies upon annealing; however, this issue needs further investigations.

**Fig. 5:**
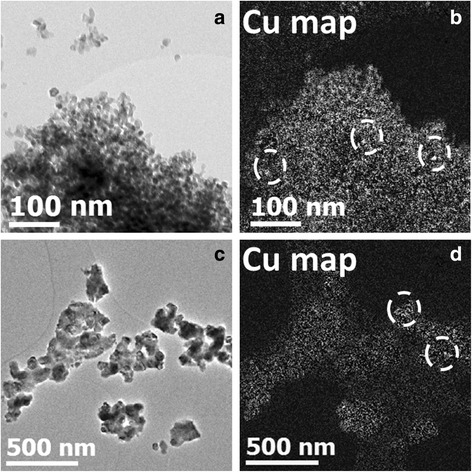
Unfiltered images (**a**, **c**) and copper maps (EFTEM images) (**b**, **d**) using the Cu M threshold energy for Cu-8 samples calcinated at *T*_c_ = 600 °C (**a**, **b**) and at *T*_c_ = 900 °C (**c**, **d**)

Chemical composition analyses have also been performed by EFTEM method to investigate the copper distribution in the samples calcinated at *T*_C_ = 600 °C (Fig. 5a, b) and 900 °C (Fig. 5c, d). Figure 5a, c is unfiltered images and Fig. 5b, d is Cu chemical maps obtained from the M energy threshold of copper. Note the different scales for both sets of images due to the significant difference in grain size. Overall, we notice a rather homogeneous distribution of copper in both cases despite the presence of bright spots suggesting some diffusion and segregation of copper in the powder.

**Fig. 6:**
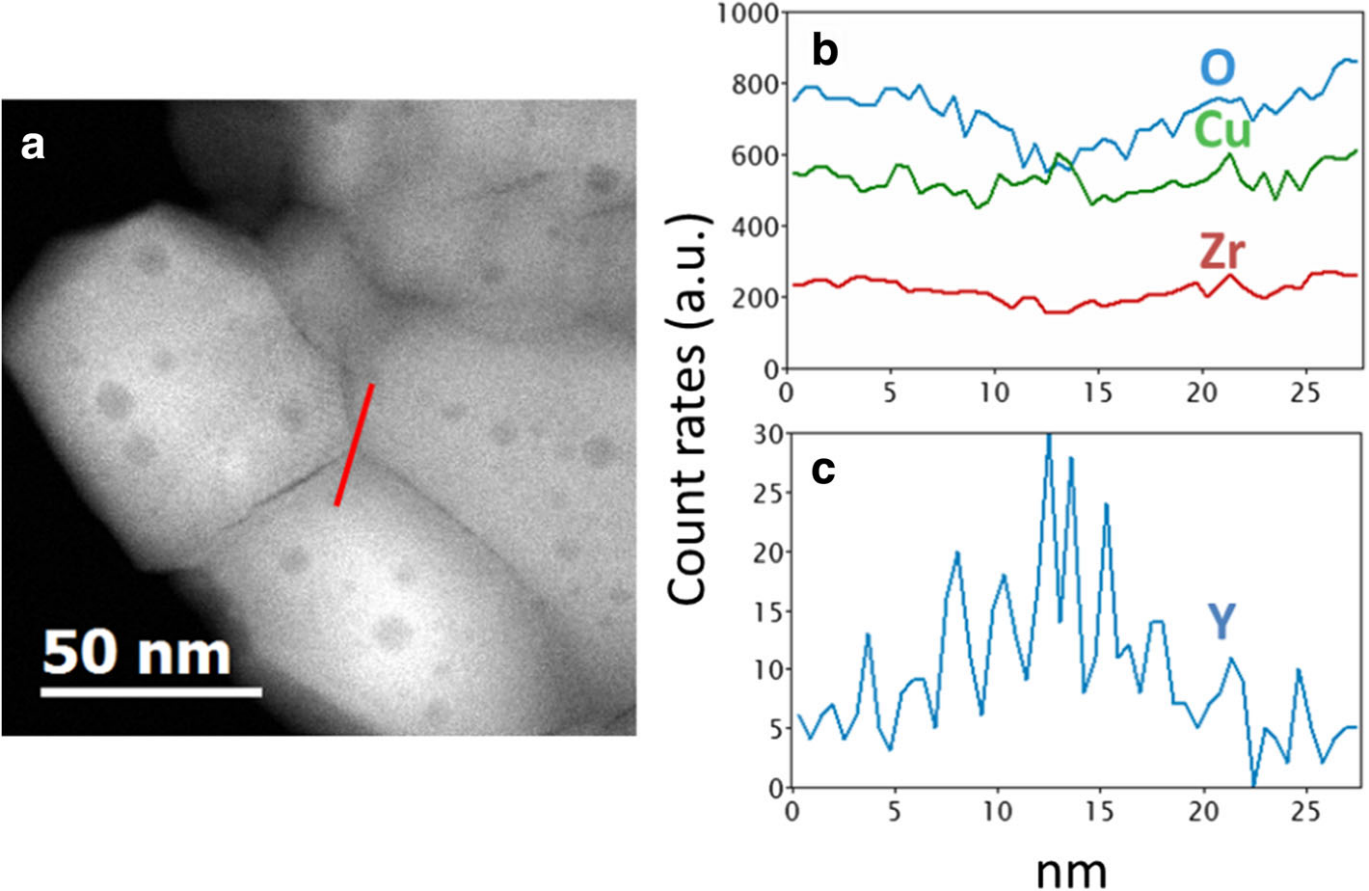
**a** STEM HAADF image of Cu-8 powder calcinated at 900 °C. The dark regions with circular shapes in the grains correspond to the cavities. **b, c** EDX profiles accross the grain boundary (*red line in* **a**) for O, Cu, and Zr (**b**) and Y (**﻿c﻿**) elements. A depletion of the number of counts for O, Cu, and Zr and an increase for Y is clearly seen

More interestingly, for higher *T*_C_, the segregations of Y and Cu at grain boundaries were observed. As one can see from Fig. 6, the STEM EDX analysis of a grain boundary (linescan along the red line in Fig. 6a) for the Cu-8 sample calcinated at 900 °C shows that these grain boundaries are enriched in Y and Cu (Fig. 6b, c).

Thus, TEM study shows that the *T*_C_ increase favors the formation of nanocrystals and their sintering at high *T*_C_ as well as the segregations of Y and Cu at grain boundaries.

### EPR Spectra

**Fig. 7:**
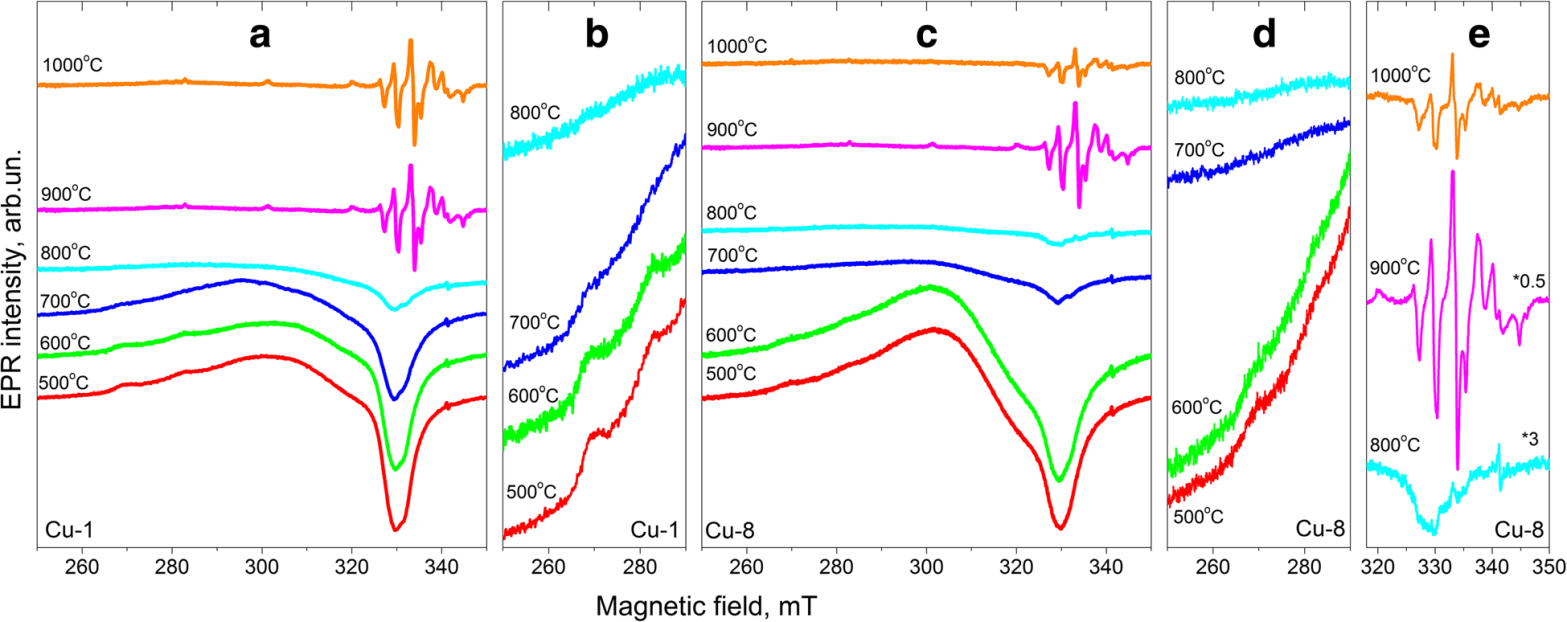
EPR spectra of Cu-1 (**a**, **b**) and Cu-8 (**c**–**e**) samples calcinated at 500–1000 °C. The spectra of the samples calcinated at *T*_c_ = 500–800 °C are denoted as “EPR spectrum I” type and others as “EPR spectrum II” type

EPR spectra of studied samples are shown in Fig. 7. Spectra of Cu-1 and Cu-8 samples annealed at the same temperature are similar. Depending on *T*_c_, there are two types of EPR spectra in our samples. The first type (denoted below as spectrum I) is observed in the samples calcinated at *T*_c_ = 500–800 °C. Another EPR spectrum (denoted below as spectrum II), contained a set of irregular shape lines in wide range of magnetic fields, is detected in the samples calcinated at 800–1000 °C (Fig. 7).

### EPR Spectrum I

**Fig. 8:**
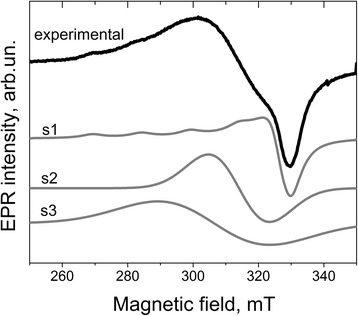
Decomposition of experimental spectrum I of Cu-8 sample calcinated at 500 °C on the components

The integral intensity of spectrum I for Cu-1 and Cu-8 samples is nearly the same. Analysis of EPR spectra of different samples shows that spectrum I consists of, at least, three signals, whose intensities depend on *T*_c_. As an example, Fig. 8 presents the decomposition of EPR spectrum of Cu-8 sample calcinated at 500 °C on the components.

**Fig. 9:**
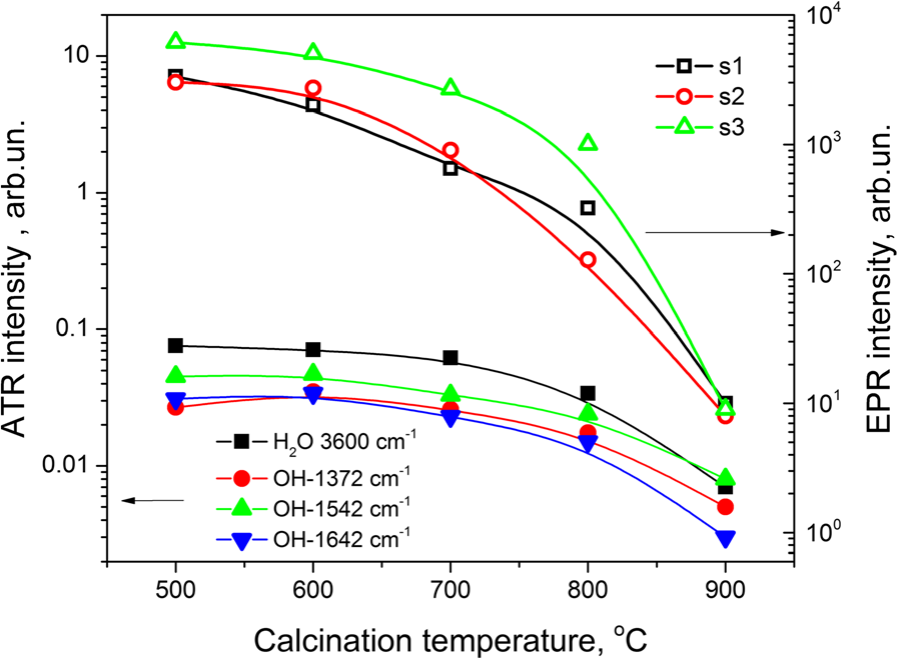
Variation of the intensities of the components of signal I (*right y-axis*) and ATR intensities of infrared absorption bands of water molecules and OH groups (*left y-axis*) on the calcination temperature for the Cu-8 samples

The first component (s1) exhibits the characteristic copper hyperfine splitting and can be described by spin-Hamiltonian parameters g_⊥_ = 2.072, g_||_ = 2.32, A_⊥_ ~ 0 G, and A_||_ ~ 150 G. Two others (s2 and s3) are single structureless lines with g ~ 2.20 and g ~ 2.15, respectively. Because these signals are absent in Cu-free samples, it can be assumed that they are also caused by Cu-related centers. In this case, the absence of Cu-related hyperfine structure can be explained by the exchange interaction between copper ions. With *T*_c_ increase, the intensities of all signals of the spectrum I decrease monotonically (Fig. 9).

The EPR spectra, similar to the spectrum I, were observed earlier in ZrO_2_ doped with copper by impregnation [23, 38, 39]. Observed spectra were considered as a superposition of at least two overlapping components. One of them, with the spin-Hamiltonian parameters g_||_ = 2.38, g_⊥_ = 2.04, A_||_ = 110 G, and A_⊥_ = 30–35 G, had resolved hyperfine structure and was attributed to isolated Cu^2+^ ions in an axially symmetrical surrounding [23, 38]. In Ref. [38], this signal was associated with the copper ions incorporated into zirconium surface vacancies and capped by oxygen atoms (i.e., with CuO molecules tightly bonded to the nanocrystals). Another signal was a single line with g = 2.23, gradually broadened with increasing of Cu content. This signal was attributed to interacting Cu^2+^ ions of neighboring CuO molecules [23, 38].

Similar EPR spectra were also observed in other copper-doped oxides (TiO_2_, ZnO, etc.). They were attributed to copper ions in the Cu-related surface complexes, in which copper ions are in the tetragonal-distorted octahedral fields of ligands [40]. In this case, the parameters of EPR signal (g-factor, hyperfine interaction, and anisotropy) were shown to be dependent on the structure of complex and type of ligands. H_2_O, O^−^, SO_4_^2−^, and (OH)^−^ were considered as possible components of complexes [39]. Specifically, the signal with spin-Hamiltonian parameters g_||_ = 2.40–2.44 and A_||_ ~ 110 G observed in Cu-doped TiO_2_ has been attributed to the copper linked with H_2_O or O_2_^-^ while the signal with g_||_ = 2.32 and A_||_ ~ 154 G has been assigned to Cu^2+^ ion associated with (OH)^−^. The latter parameters are close to the parameters of the s1 component. Therefore, this signal is more likely related to the surface complex containing OH groups. This statement is supported by the decrease of its intensity with the decrease in the amount of water molecules and OH groups at the surface of the nanocrystals with *T*_c_ (Fig. 9). Such dependence is also observed for other components of spectrum I.

**Fig. 10:**
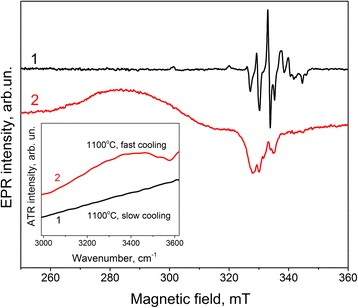
EPR spectra of Cu-8 sample calcinated at 1100 °C followed by slow cooling (*curve 1*) or by quenching (*curve 2*). The corresponding infrared absorption spectra are shown in the *inset*

Correlation between this EPR spectrum and the presence of surface complexes involving water molecules and/or OH groups is also evidenced by an annealing of the xerogel at 1100 °C followed by quenching. This treatment results in the simultaneous appearance of EPR spectrum I and infrared absorption bands related to OH groups and H_2_O molecules (Fig. 10). The same annealing followed by slow cooling does not cause either infrared absorption bands or EPR spectrum I.

Thus, the EPR spectrum I can be attributed to the surface complexes containing copper ions linked with water molecules and/or OH groups. It should be noted that Cu-related complexes with different ligands (NH_3_, OH, H_2_O) were observed in Cu–Y–Zr–O hydrogel [41].

Note that the decrease of EPR intensity of spectrum I is accompanied by the increase of the absorption band at 600–900 nm related to surface copper ions (either Cu from CuO molecules or Cu_Zr_ substitutions). Since *T*_c_ increase above 800 °C results in the transformation of this absorption band towards that of crystalline CuO, one can ascribed absorption band observed for samples calcinated at *T*_c_ = 500–800 °C to dispersed CuO. In this case, the anti-correlation between the intensities of EPR spectrum I and the absorption band at 600–900 nm does not allow explaining this EPR signal by CuO molecules or clusters as proposed in [23, 38, 39]. Besides, in spite of the same intensities of EPR signal I in Cu-1 and Cu-8 samples (Fig. 7), the intensity of CuO absorption band is higher for the Cu-8 samples (Fig. 2). This means that dispersed and crystalline CuO are non-paramagnetic.

### EPR Spectrum II

As it was mentioned above, this spectrum is observed in the samples calcinated at higher (800–1000 °C) temperatures. It is a set of irregular shape lines in wide range of magnetic fields. The narrow EPR lines (and therefore the absence of spin-Hamiltonian parameters distribution) indicate that the paramagnetic centers responsible for this spectrum are in regular positions with stable surrounding. Besides, the presence of characteristic hyperfine lines allows reasonable assuming that this spectrum or at least its main part is caused by substitutional Cu^2+^ ions (Cu_Zr_).

**Fig. 11:**
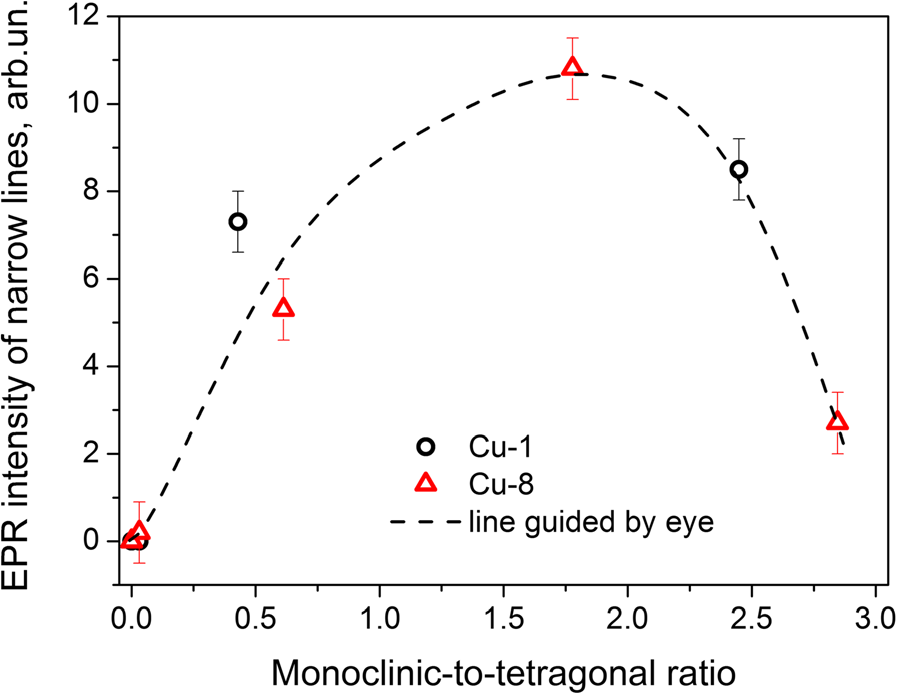
Variation of the intensity of EPR signal II vs. monoclinic-to-tetragonal (-cubic) ratio for Cu-1 (*circles*) and Cu-8 (*triangles*) samples

For both types of samples (Cu-1 and Cu-8 samples), signal II shows different behaviors with temperature increase (Fig. 7), but it has similar trend with the contribution of monoclinic phase: its intensity initially increases with monoclinic-to-tetragonal (-cubic) ratio and then decreases (Fig. 11). Since the signal II is observed only when monoclinic phase appears, it can be assumed to be caused by Cu_Zr_^2+^ ions in monoclinic structure. The absence of signal of Cu_Zr_^2+^ ions in tetragonal structure can be related, for example, with rapid spin relaxation processes.

### Copper Redistribution and Phase Transformation

The above results provide information about the redistribution of copper between the volume and the surface of the nanocrystals according to the calcination temperature. As it was noted above, in diffuse reflectance spectra of the samples calcinated at *T*_c_ = 500 °C, the absorption band at ~270 nm caused by oxygen vacancies is observed. Its intensity is higher in Cu-8 samples than that in Cu-1 ones and substantially exceeds the intensity in the Cu-free Y-doped ZrO_2_ powders (Fig. 2).

It is obvious that the presence of yttrium already creates oxygen vacancies in ZrO_2_. However, the Cu doping increases their number due to the higher difference in the valencies of Cu and Zr ions. Higher intensity of the absorption peak at ~270 nm in Cu-8 samples in comparison with that in Cu-1 ones is caused by higher Cu content in nanocrystal volume of corresponding samples.

The presence of copper in the bulk of nanocrystals was also confirmed by XRD data [22, 31]. The latter showed the shift of XRD peak positions to higher diffraction angles in comparison with those of Cu-free Y-doped ZrO_2_ samples. This shift is more pronounced for Cu-8 samples.

With *T*_c_ rise (in the range of 500–700 °C) for Cu-8 samples, the absorption band at ~270 nm intensifies indicating an increase in the number of oxygen vacancies in the nanocrystals. It testifies to the enrichment of nanocrystal volume with copper that is also confirmed by additional shift of XRD peak positions to higher angles [22, 31].

This can be assigned to Cu in-diffusion from the Cu-related surface complexes observed in EPR spectra. Indeed, the *T*_c_ increase leads to the decrease of corresponding EPR signal intensity due to destruction of these complexes as a result of water or OH group loss (Fig. 9). Simultaneously, the intensity of CuO-related absorption band (in the range of 600–900 nm) increases. This finding can be explained by the following: part of Cu^2+^ ions appeared due to destruction of surface complexes incorporates additionally into nanocrystals volume, while another one is oxidized forming CuO molecules.

As *T*_c_ increases in the range of 800–1000 °C, the intensity of the band at ~270 nm reduces. This is consistent with the appearance of monoclinic phase and its increasing contribution (Fig. 1) that is confirmed by ATR spectra (Fig. 1) and XRD data [22, 31]. The appearance of the monoclinic ZrO_2_ phase (Fig. 1) can be explained by outward diffusion of Cu which stimulates outward diffusion of Y from such grains [32]. The Cu out-diffusion is confirmed by the enhancement and high-energy shift of the absorption band related with CuO molecules as well as by the appearance of the absorption feature of crystalline CuO (Fig. 2). This decrease of Cu and Y contents in nanocrystals has been observed by EDX method proving consequently their segregation at grain boundaries (Fig. 6). Additional argument for Cu out-diffusion is the shift of XRD peak positions of tetragonal nanocrystals to lower angles [31]. Such phase transformation results in the non-uniform distribution of dopants at a nanometer scale in the powders. Since all the grains were found to contain Cu, one can suppose that the surface of, at least, monoclinic grains are covered by CuO.

The Cu relocation can also explain the non-monotonic dependence of the EPR spectrum II intensity vs. ratio of monoclinic to tetragonal (cubic) phases. In fact, the increase of the signal intensity with this ratio can be caused by the increasing number of nanocrystals with monoclinic structure, while the decreasing of EPR signal II intensity can be assigned to copper out-diffusion from nanocrystals volume.

Thus, coprecipitation method allows obtaining nanocomposite with copper on the surface and inside of nanocrystals. Variation of calcination temperature can change the copper concentration on the surface and in the volume of nanocrystals, as well as transformation of Cu-related surface entities. Indeed, besides CuO molecules, complexes containing copper ions, water molecules, or OH group are present in the samples calcinated at *T*_c_ = 500–800 °C. These complexes are destructed with the *T*_c_ increasing. At higher calcination temperatures, the dispersed or crystalline CuO dominates.

## Conclusions

The influence of calcination temperature on copper localization in ZrO_2_ composites doped with Y and Cu is studied by ATR, diffuse reflectance, and EPR techniques. The rise of calcination temperature in the range of 500–700 °C results in the increase of the intensity of the absorption band peaked at ~270 nm. This band is assumed to be caused by oxygen vacancies in the nanocrystals, and its enhancement is explained by an increase of copper concentration in nanocrystal volume. The latter occurs due to Cu incorporation from the surface, in particular, from the surface complexes observed in the EPR spectra. It is shown that these complexes include copper ions and water molecules or OH groups and are destroyed with the temperature growth in the range of 500–700 °C.

The *T*_c_ increase in the 800–1000 °C range reduces the copper concentration in the nanocrystals’ volume due to its outward diffusion. This results in the quenching of ~270-nm absorption band, the appearance of the monoclinic phase, and the increase of light absorption by dispersed and crystalline CuO appeared in the 600–900 nm spectral range.

## Abbreviations

ATR: Attenuated total reflection
DR: Diffused reflectance
EDX: Energy dispersed X-ray spectroscopy
EPR: Electron paramagnetic resonance
TEM: Transmission electron microscopy
XRD: X-ray diffraction
